# Memory effects and self-excited oscillations in deterministic epidemic models with intrinsic time delays

**DOI:** 10.1140/epjp/s13360-020-00862-2

**Published:** 2020-10-27

**Authors:** R. De Luca, F. Romeo

**Affiliations:** Dipartimento di Fisica “E. R. Caianiello”, Università degli Studi di Salerno, 84084 Fisciano, SA Italy

## Abstract

The simplest delay differential equation describing the dynamics of non-lethal infectious diseases in a fixed-size population is extended to include the incubation period, as an additional delay parameter. It is observed that these types of deterministic models consist of one delay differential equation, whereas standard SIR and SEIR models consist of two and three ordinary differential equations, respectively. The extended model presents interesting peculiarities as, for example, initial oscillatory patterns in the curve counting the infectious individuals. A comparison of the doubly delayed differential equation with the standard SEIR model is made. It is argued that self-sustained oscillations, which are intrinsic properties of models with time delay, have to be taken into account in designing optimal epidemic containment strategies.

## Introduction

Recently, due to the pandemic outbreak of COVID-19 [1], infectious disease dynamics has become an important topic. Decision makers, journalists, and ordinary people have been exposed to the precise terminology developed during the years to describe the results obtained by mathematical models dedicated to the way infectious diseases spread in a closed population. A rather exhaustive essay on deterministic epidemiological models has been given by Hethcote [2]. Stochastic [3, 4] and network [5] approaches are also important in providing a more detailed description of the individual interactions. All these classes of models are to be considered, in order to acquire a complete picture of spatial and time dependence of observables, like the number of susceptible, exposed, infectious, or recovered individuals, during an infectious disease outbreak. Compartmental deterministic models, like SIR and SEIR models and their extensions [2, 4], are particularly suitable to study optimal strategies to mitigate the epidemic evolution, and thus they are frequently mentioned in current mass media debates.

In the last 10 years, there has been a growing interest in epidemiological models, considering the paramount importance of the sanitary problems related to epidemic outbreaks. These models have been developed also with the intent of addressing different aspects of infectious disease dynamics, such as temporary immunity and possible secondary infection [6], effectiveness of vaccination strategies [7], seasonal character of epidemic outbreaks [8], and controllability of chaotic response [9].

As for deterministic mathematical models describing the dynamics of infectious diseases in a closed population, they are commonly classified using the acronyms SIR and SEIR [10–13]. In the second class of models, individuals are partitioned in compartments collecting susceptible ($S$), exposed ($E$), infectious ($I$), and recovered ($R$) individuals; in the first, the exposed ($E$) class, collecting infected individuals not yet infectious, is not taken in account.

On the other hand, a rather different formulation of the epidemic evolution can be expressed in terms of delay differential equations. The use of delay differential equations in epidemiology dates back to the pioneering work of Van Der Plank [14] who introduced these models to describe plant diseases. Van Der Plank work had a limited impact in the context of human/animal diseases models, probably because the model assumptions are very specific of plant pathologies. However, in Ref. [15], it has been demonstrated that a variant of Van Der Plank model is perfectly suited to describe human/animal diseases. In particular, the Noviello–Romeo–De Luca (NRD) model [15] consists of a single delay differential equation (DDE). The latter model describes, much in the same way as the SIR model does, the infectious disease dynamics in the absence of an incubation period. Nonetheless, the incubation period is an essential ingredient in achieving a correct description, prevention, and control of specific illnesses [16]. Moreover, introduction of this parameter in the model improves the understanding of disease dynamics.

To add this ingredient, in the present work we extend the NRD model to the case in which a constant quiescence time $t_{I}$ is present in the system. Differently from the existing models [17] based on DDE, we look for a minimal model, which is here denoted as NRDE model. Thus, in the following, we provide a detailed derivation of the NRDE model also discussing the effects on the dynamics of the two time delays $t_{R}$ and $t_{I}$.

Therefore, the paper is organized as follows. In Sect. 2 we review the SIR, SEIR, and NRD models. In Sect. 3 we construct the NRDE model by including an additional delay time $t_{I}$ in the NRD model. A comparison between SEIR and NRDE models is made in Sect. 4. Oscillatory patterns in the NRDE model are studied in Sect. 5. Finally, conclusions are drawn in the last section.

## The SIR, SEIR, and NRD models

Let us now first consider the SIR and SEIR models. Denoting by $S\left( t \right)$, $E\left( t \right)$, $I\left( t \right)$, and $R\left( t \right)$ the number of individuals belonging to the classes $S$, $E$, $I$, and $R$, respectively, at time $t$, by the closed population assumption (absence of vital dynamics) one can write the following constraint:

1 $$ S\left( t \right) + E\left( t \right) + I\left( t \right) + R\left( t \right) = N . $$

Assuming validity of the prey–predator interaction between susceptible and infectious individuals, the SEIR model can be summarized by the following set of differential equations:

2a $$ \frac{\text{d}}{{{\text{d}}t}}S\left( t \right) = - \frac{\beta }{N}S\left( t \right)I\left( t \right) , $$

2b $$ \frac{\text{d}}{{{\text{d}}t}}E\left( t \right) = \frac{\beta }{N}S\left( t \right)I\left( t \right) - \delta E\left( t \right), $$

2c $$ \frac{\text{d}}{{{\text{d}}t}}I\left( t \right) = \delta E\left( t \right) - \gamma I\left( t \right), $$

where the parameters $\beta$, $t_{R} = \frac{1}{\gamma }$, and $t_{I} = \frac{1}{\delta }$, are the effective infection rate, the average recovery time, and the average duration of the latent period, respectively. Naturally, because of Eq. (1), it is not necessary to exhibit the fourth differential equation $\frac{\text{d}}{{{\text{d}}t}}R\left( t \right) = \gamma I\left( t \right)$ for the model.

The SIR model can be obtained disregarding the exposed class. In the latter model, by describing the initial part of the time evolution of the curve $I\left( t \right)$, the value of the basic reproduction number $R_{0} = \frac{\beta }{\gamma }$ can be associated to two different dynamic regimes of the system. In fact, in an initial interval of time, where $S\left( t \right) \approx N$, the number of infectious individuals is determined by the following differential equation:

3 $$ \frac{\text{d}}{{{\text{d}}t}}I\left( t \right) = \left( {R_{0} - 1} \right)\gamma I\left( t \right), $$

so that the relations $R_{0} > 1$ or $R_{0} < 1$ imply initial exponential growth or decay of $I\left( t \right)$, respectively, while $R_{0} = 1$ implies that the number of infectious individuals is left initially unaltered.

In the NRD model [15], which is the simplest epidemic model with time delay, it is necessary to introduce the cumulative function

4 $$ M\left( t \right) = I\left( t \right) + R\left( t \right), $$

equivalent to counting all individuals being or having been ill. In this SIR-like model, with the aid of the average recovery time $t_{R}$, the functions $S\left( t \right)$, $I\left( t \right)$, and $R\left( t \right)$ can be expressed as follows:

5a $$ S\left( t \right) = N - M\left( t \right), $$

5b $$ I\left( t \right) = M\left( t \right) - M\left( {t - t_{R} } \right), $$

5c $$ R\left( t \right) = M\left( {t - t_{R} } \right). $$

Therefore, by substituting in Eq. (2a) the expressions for $S\left( t \right)$ and $I\left( t \right)$ given in Eqs. (5a) and (5b), respectively, the model is represented by the following delay differential equation (DDE):

6 $$ \frac{\text{d}}{{{\text{d}}t}}M\left( t \right) = \frac{\beta }{N}\left[ {N - M\left( t \right)} \right]\left[ {M\left( t \right) - M\left( {t - t_{R} } \right)} \right] . $$

The above DDE requires knowledge of the cumulative function $M\left( t \right)$ in the time interval $\left[ { - t_{R} ,0} \right]$. In this respect, the model keeps memory of the history of the function $M\left( t \right)$. In Ref. [15], however, the following simple choice for the function $M\left( t \right)$ in the time interval $\left[ { - t_{R} ,0} \right]$ has been made:

7 $$ M\left( t \right) = \left\{ {\begin{array}{*{20}l} 0 \hfill &\quad {{\text{for}}\, - t_{R} \le t < 0} \hfill \\ {M_{0} = I_{0} = N - S_{0}  } \hfill &\quad {{\text{for}}\, t = 0} \hfill \\ \end{array} } \right. . $$

## The NRDE model

In Ref. [15], it has been proven that the quantity $t_{R}$ represents a natural time scale and appears as a constant delay time in the nonlinear differential equation defining the NRD model. In fact, starting from a semi-continuous analysis of the problem, the authors have shown that a single globally defined function $M\left( t \right)$ can be adopted to describe the dynamics of all classes of individuals, namely, $S$, $I$, and $R$. The NRD model is similar to the SIR model, sharing with it the concept of the basic reproduction number $R_{0}$. Furthermore, the initial conditions in the SIR model can be emulated as in Eq. (7). In addition, as for the outcome of the NRD model, a characteristic *S*-shaped curve for the number of recovered individuals $R\left( t \right)$, similar to the one obtained for the SIR model, gives the same asymptotic value $R_{\infty }$ for $t \to + \infty$. Both models predict extinction of the illness after a sufficiently long time, with the same regimes indicated for the SIR model in Sect. 2. Moreover, in the epidemic regime, the time evolution of the number of infectious individuals $I\left( t \right)$ presents an initial exponential increase, a central peak value and a subsequent decrease to zero. The same behavior can be found in the corresponding curves obtained by means of the SIR model.

Both SIR and NRD models cannot account for a possible quiescent time of the illness, i.e., a period in which an infected individual is not yet infectious. Therefore, an extended NRD model (NRDE model) needs to be introduced in the same way a SEIR model is developed starting from a SIR model. In order to develop an extended model, two time delays, i.e., $t_{R}$ and $t_{I}$, need to be considered. Investigation of the dynamic interplay between these two intrinsic time scales of the system constitutes the main purpose of this work.

In this section, we provide the fundamental time delay equation describing the dynamics of non-lethal infectious diseases with incubation time $t_{I}$. We shall show that the NRDE model reduces to the NRD model by simply setting $t_{I} = 0$, since the latter time is naturally conceived as an additional delay in the model. In order to establish this point, we notice that the recovery time represents the delay with which a susceptible individual from $S$ reaches class $R$ in the NRD model, passing through class $I$, there remaining for an average time $t_{R}$. In this way, by use of $M\left( t \right)$, one can write Eq. (5b) as the difference of two shifted values of the same cumulative function.

We start by assuming that it is not possible to detect the illness, unless the individual is infectious, so that individual belonging to class $E$ cannot be detected as such until they make a transition to class $I$. Therefore, the cumulative function can be still defined as in Eq. (4). However, we need to modify Eqs. (5a–c) and write and additional expression for $E\left( t \right)$. For this purpose, we may notice that the expression for the number of infectious and recovered individuals are unaltered, because of the starting assumption that individuals in class $E$ are not infectious and can be detected only when they make a transition in the class $I$. For the same reason, in order to determine the number of susceptible individuals at time $t$, we need to subtract from the total population number $N$ all the individuals in the other classes. Therefore, since $M\left( t \right)$ counts only the infected and recovered individuals at time $t$, we may add to this number the function $E\left( t \right)$ by considering all medical records at $t + t_{I}$. In this way, we end up with the following relations:

8a $$ S\left( t \right) = N - M\left( t \right) - E\left( t \right) $$

8b $$ M\left( t \right) + E\left( t \right) = M\left( {t + t_{I} } \right) $$

By summarizing the dependence of all functions $S\left( t \right)$, $E\left( t \right)$, $I\left( t \right)$, and $R\left( t \right)$ on $M\left( t \right)$, we write:

9a $$ S\left( t \right) = N - M\left( {t + t_{I} } \right), $$

9b $$ E\left( t \right) = M\left( {t + t_{I} } \right) - M\left( t \right), $$

9c $$ I\left( t \right) = M\left( t \right) - M\left( {t - t_{R} } \right), $$

9d $$ R\left( t \right) = M\left( {t - t_{R} } \right). $$

Having defined the way the number of individuals can be expressed in terms of the cumulative function $M\left( t \right)$, we may specify the history of the disease for $t \in \left[ { - t_{R} ,0} \right]$. In order to mimic the initial conditions of a SEIR model, for example, we may adopt the same history described by Eq. (7). However, we remark that the initial history may also play a role in determining the dynamics of the system and thus this issue deserves further consideration. Before writing down the dynamic equation for the NRDE model, let us make the following time transformation:

10 $$ t^{\prime } = t + t_{I} . $$

In this way, we first write Eq. (2a) in terms of the new time variable $t^{\prime}$, by writing:

11 $$ \frac{\text{d}}{{{\text{d}}t^{\prime } }}S\left( {t^{\prime } } \right) = - \frac{\beta }{N}S\left( {t^{\prime } } \right)I\left( {t^{\prime } } \right) . $$

By now considering Eqs. (9a), (9c), and (10), we have:

12 $$ \frac{\text{d}}{{{\text{d}}t^{\prime } }}M\left( {t^{\prime } } \right) = \frac{\beta }{N}\left[ {N - M\left( {t^{\prime } } \right)} \right]\left[ {M\left( {t^{\prime } - t_{I} } \right) - M\left( {t^{\prime } - t_{I} - t_{R} } \right)} \right] . $$

The history (7) now becomes:

13 $$ M\left( {t^{\prime } } \right) = \left\{ {\begin{array}{*{20}l} 0 \hfill & {{\text{for}}\, - \left( {t_{R} + t_{I} } \right) \le t^{\prime } < 0} \hfill \\ {M_{0} = I_{0} = N - S_{0} } \hfill & {{\text{for}}\, t^{\prime } = 0} \hfill \\ \end{array} } \right.. $$

Let us now define the following dimensionless quantities:

14 $$ \tau = \frac{{t^{\prime } }}{{t_{R} }};\tau_{I} = \frac{{t_{I} }}{{t_{R} }}; s\left( \tau \right) = \frac{S\left( \tau \right)}{N}; e\left( \tau \right) = \frac{E\left( \tau \right)}{N}; i\left( \tau \right) = \frac{I\left( \tau \right)}{N}; r\left( \tau \right) = \frac{R\left( \tau \right)}{N}, m\left( \tau \right) = \frac{M\left( \tau \right)}{N}, $$

where the time $\tau$ is measured in units of $t_{R}$ and the number of individuals $X\left( \tau \right)$ in the generic class $X$ is expressed more conveniently in terms of the ratio $0 \le x\left( \tau \right) = \frac{X\left( \tau \right)}{N} \le 1$. Because of Eqs. (14), (12) and (13) can be written as follows:

15a $$ \frac{\text{d}}{{{\text{d}}\tau }}m\left( \tau \right) = R_{0} \left[ {1 - m\left( \tau \right)} \right]\left[ {m\left( {\tau - \tau_{I} } \right) - m\left( {\tau - \tau_{I} - 1} \right)} \right],\,(\tau > 0), $$

15b $$ \begin{array}{*{20}l} {m\left( \tau \right) = 0   } \hfill & {{\text{for }}\,\tau \in \left[ { - \left( {1 + \tau_{I} } \right),0} \right[} \hfill \\ {m\left( \tau \right) = m_{0} = 1 - s_{0} } \hfill & {{\text{for }}\,\tau = 0} \hfill \\ \end{array} , $$

where $$ m_{0} = \frac{{M_{0} }}{N} $$ and $$ s_{0} = \frac{{S_{0} }}{N} $$.

Furthermore, because of Eqs. (10) and (14), the expressions in Eqs. (9a–d) can be written as follows:

16a $$ s\left( \tau \right) = 1 - m\left( {\tau + \tau_{I} } \right), $$

16b $$ e\left( \tau \right) = m\left( {\tau + \tau_{I} } \right) - m\left( \tau \right), $$

16c $$ i\left( \tau \right) = m\left( \tau \right) - m\left( {\tau - 1} \right), $$

16d $$ r\left( \tau \right) = m\left( {\tau - 1} \right) . $$

Equation (15a, b) and (16a–d) completely define the NRDE model. Examining the main dynamic features of the model, we may observe that, as in the NRD model, a single DDE is sufficient to describe the time evolution of the number of individuals in the various classes, as specified in Eqs. (16a–d). The dynamics is expressed in terms of the same cumulative function $m\left( \tau \right)$ encountered in the NRD model. From Eq. (15a), we may notice that this function is forced to be a monotonically increasing function. Moreover, $m\left( \tau \right)$ is also a non-negative bounded function. Finally, examining Eq. (15a), one can observe that time evolution of the system, depending on the specific initial history, may proceed toward two distinct fixed points which are reached when either the number of susceptible individuals or the number of infectious individuals is equal to zero. In this way, $m\left( \tau \right)$ cannot show chaos, i.e., long-term aperiodic behavior [18]. For these reasons the dynamics of the NRDE model, although presenting peculiar and extremely important fingerprints discussed in the following section, does not show qualitative differences from the models reviewed in Sect. 3. However, if a time-discretized version of the model would be developed, starting from Eq. (15a), chaotic behavior could not be excluded. The latter analysis is beyond the purpose of the present work.

It is worth mentioning that this model shares the same type of limitations of many deterministic compartmental models like, for instance, the SIR and NRD models. In fact, just to mention a not exhaustive list of limitations, the model disregards vital dynamics, age-stratified incidence of the disease, detailed diffusion mechanisms on the social network, and seasonal effects. Despite these limitations, the model presents an intrinsic dynamic richness and can be further extended to include some of these features. For example, in order to take account of the age-stratified incidence of the disease, an NRDE model with age-dependent recovery time can be developed.

## Comparison between SEIR and NRDE models

The NRDE model relies upon a single time-delay differential equation, namely, Eq. (15a), and reduces to the NRD model for $\tau_{I} = 0$. In the present section we shall look for similarities and differences between the SEIR and the NRDE models. In fact, the NRDE model can be considered the time-delayed version of a SEIR model. For this reason, a comparison between SEIR and NRDE dynamics is appropriate. For this purpose, we shall first investigate the dynamic properties of the curves $i\left( \tau \right)$ and $r\left( \tau \right)$; successively, we shall take a close look at the duration $\tau_{D}$ of the disease.

Standard numerical routines are used to simulate the time evolution of the observables $i\left( \tau \right)$ and $r\left( \tau \right)$, as outcome of the SEIR and NRDE models, starting from a value of $m_{0} = 0.005$ ($s_{0} = 0.995$) and $R_{0} = 2.50$ to fix our ideas.

**Fig. 1:**
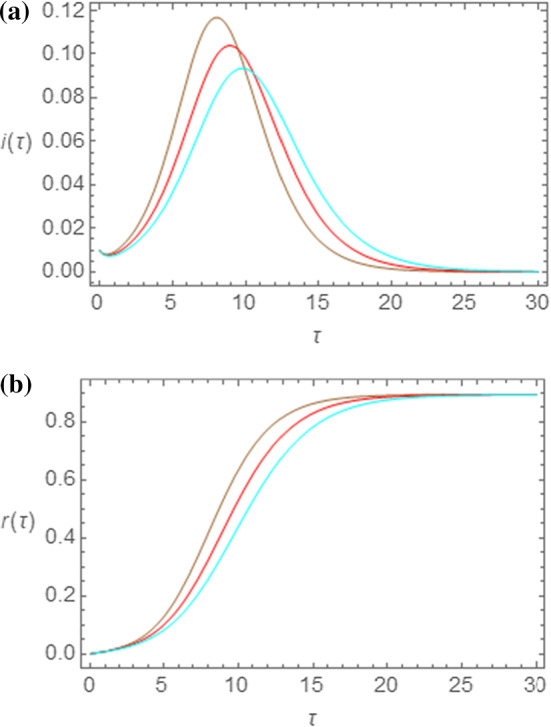
Outcome of the SEIR model for $s_{0} = 0.995$, $R_{0} = 2.50$ and for $\tau_{I} = 1.00$ (brown curves), $\tau_{I} = 1.25$ (red curves), and $\tau_{I} = 1.50$ (cyan curves). In (**a**) and (**b**) the time evolutions of $i\left( \tau \right)$ and $r\left( \tau \right)$ are shown, respectively

In Fig. 1a, b, we show the outcome of the SEIR model for this choice of parameters $R_{0}$ and $s_{0}$ and for three different values of $\tau_{I}$, namely, $\tau_{I} = 1.00$ (brown curves), $\tau_{I} = 1.25$ (red curves), and $\tau_{I} = 1.50$ (cyan curves). In particular, in Fig. 1a the bell-shaped time dependence of the quantity $i\left( \tau \right)$ is shown. The observed trend in these curves is a gradual shift of the maximum point to the right, with a concomitant decrease of the maximum value, as $\tau_{I}$ increases. In Fig. 1b, on the other hand, the S-shaped curves for $r\left( \tau \right)$ are shown. While the asymptotic value $r_{\infty }$ of these curves remain unaltered for all values of $\tau_{I}$, the increasing ramp shifts to the right as the value of $\tau_{I}$ increases.

**Fig. 2:**
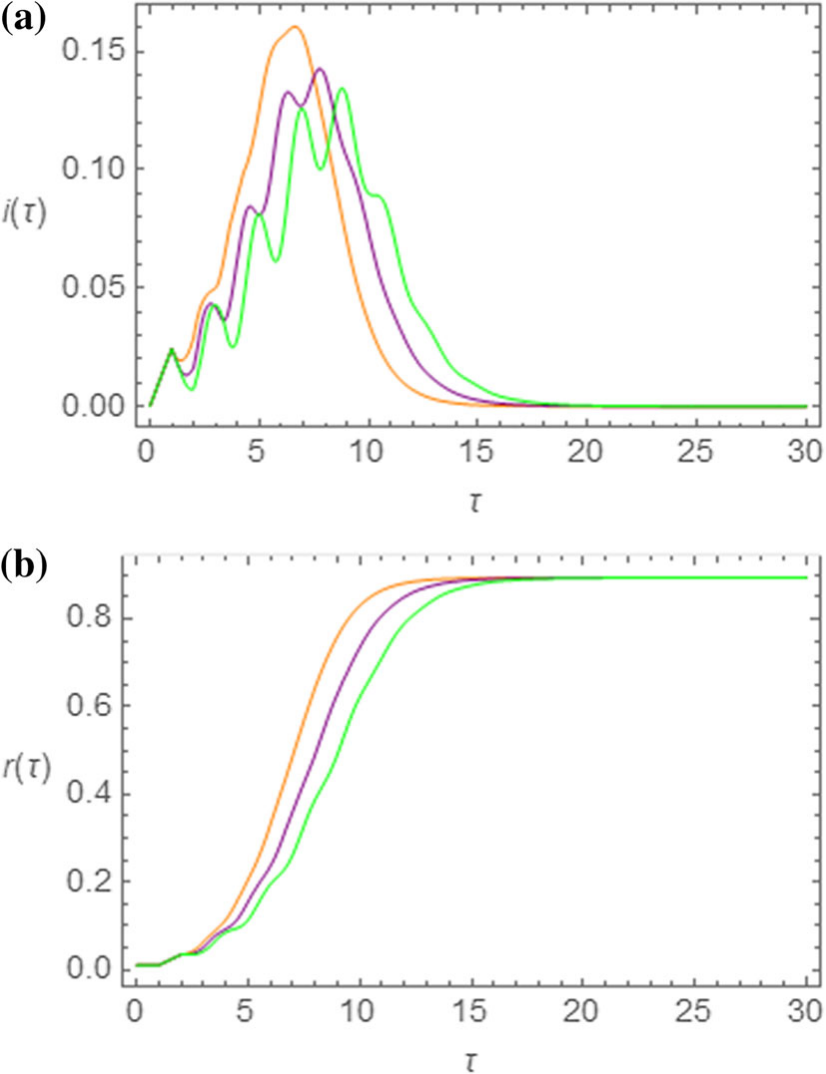
Outcome of the NRDE model for $s_{0} = 0.995$, $R_{0} = 2.50$ and for $\tau_{I} = 1.00$ (orange curves), $\tau_{I} = 1.25$ (purple curves), and $\tau_{I} = 1.50$ (green curves). In (**a**) and (**b**) the time evolutions of $i\left( \tau \right)$ and $r\left( \tau \right)$ are shown, respectively

In Fig. 2a, b we show the outcome of the NRDE model for the same choice of the parameters $R_{0}$ and $s_{0}$ and for the same three different values of $\tau_{I}$, namely, $\tau_{I} = 1.00$ (orange curves), $\tau_{I} = 1.25$ (purple curves), and $\tau_{I} = 1.50$ (green curves). In particular, in Fig. 2a, the curves for $i\left( \tau \right)$ are shown. The same qualitative features as in Fig. 1a are present in the global behavior of these curves. However, an additional oscillating behavior, superimposed to the bell profile, is detected. This type of behavior will be investigated more closely in the next section. In Fig. 2b, on the other hand, qualitatively similar S-shaped curves are reported for this model. We observe that the same asymptotic value $r_{\infty }$ is reached for all values of $\tau_{I}$, as for the SEIR model.

**Fig. 3:**
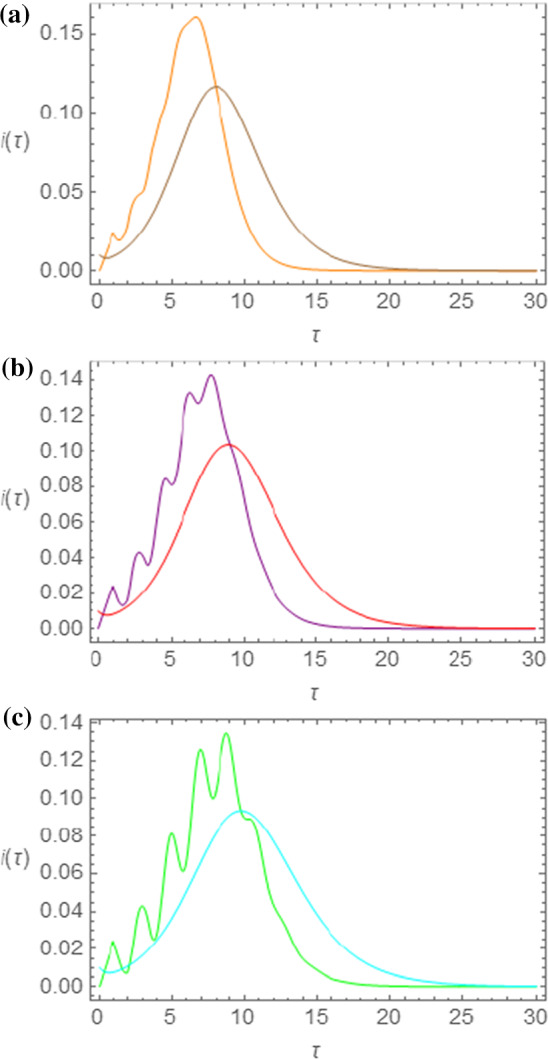
Comparison between the $i\left( \tau \right)$ curves obtained by means of the SEIR and of the NRDE models for $s_{0} = 0.995$, $R_{0} = 2.50$ and for $\tau_{I} = 1.00$ (**a**), $\tau_{I} = 1.25$ (**b**), and $\tau_{I} = 1.50$ (**c**). The same choice of colors as in Figs. 1a and 2a has been made

**Fig. 4:**
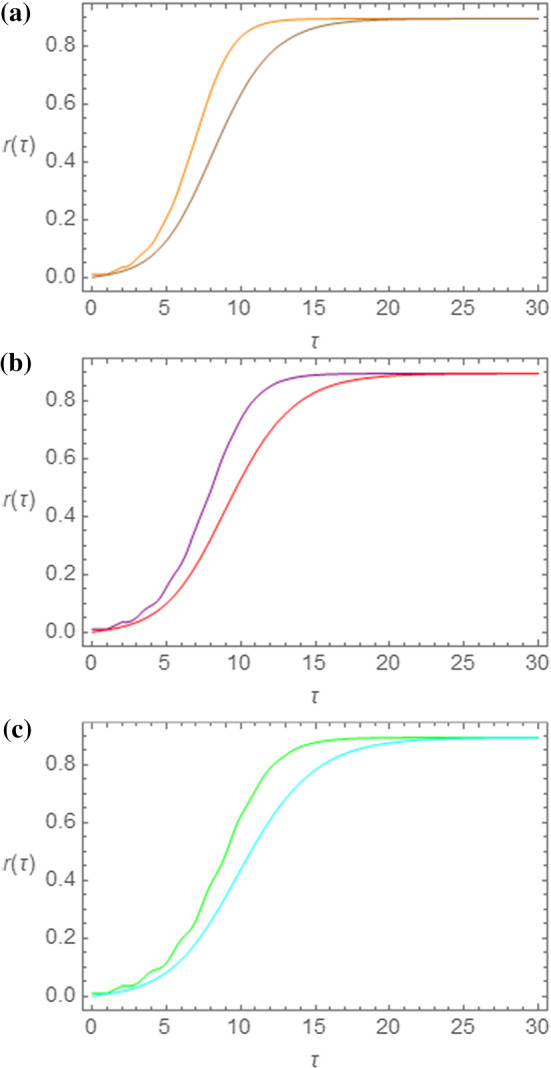
Comparison between the ${r\left( \tau \right)}$ curves obtained by means of the SEIR and of the NRDE models for $s_{0} = 0.995$, $R_{0} = 2.50$ and for $\tau_{I} = 1.00$ (**a**), $\tau_{I} = 1.25$ (**b**), and $\tau_{I} = 1.50$ (**c**). The same choice of colors as in Figs. 1b and 2b has been made

In order to make a close comparison between these two sets of curves, the results obtained, for the same choice of parameters for the SEIR and the NRDE models, are reported on the same graph in Fig. 3a–c and in Fig. 4a–c for $m_{0} = 0.005$ ($s_{0} = 0.995$) and $R_{0} = 2.50$.

Therefore, in Fig. 3a–c, we show the $i\left( \tau \right)$ curves obtained for the two models for $\tau_{I} = 1.00$ (a), $\tau_{I} = 1.25$ (b), and $\tau_{I} = 1.50$ (c). In these curves we notice that the NRDE model curves reach higher maximum values and show an increase at earlier times with respect to the SEIR curves. Thus, even though the curves are qualitatively similar, they differ on the quantitative point of view.

**Fig. 5:**
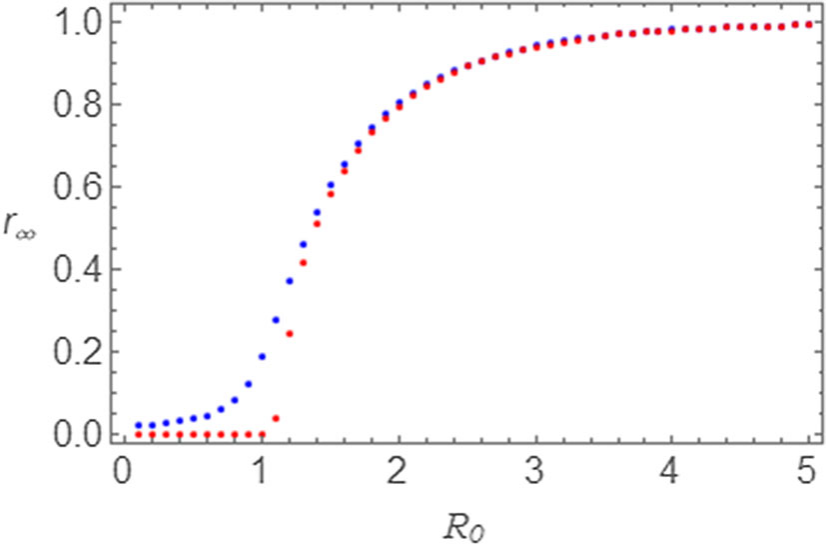
Asymptotic value $r_{\infty }$ of the curve $r\left( \tau \right)$ as a function of the parameter $R_{0}$ for $\tau_{I} = 1.0$. SEIR model: $s_{0} = 0.980$ (blue points); $s_{0} = 0.999$ (red points). The output for the NRDE model juxtapose to those obtained with the SEIR model

In Fig. 4a–c the $r\left( \tau \right)$ curves obtained for the two models for $\tau_{I} = 1.00$ (a), $\tau_{I} = 1.25$ (b), and $\tau_{I} = 1.50$ (c) are shown. Once again we note that, even though the shape of the curves is similar, the NRDE model curves show an increase at earlier times with respect to the SEIR curves. On the other hand, the asymptotic value $r_{\infty }$ reached by the curves is the same. Therefore, in the epidemic regime, the quantity $r_{\infty }$ shows independence from the model and from the parameter $\tau_{I}$. This same consideration can be extended to the SIR and NRD models, because of the independence of $r_{\infty }$ on $\tau_{I}$. We may therefore give a numerical account of this aspect, by exhibiting $r_{\infty }$ versus $R_{0}$ plots at a fixed value of $\tau_{I}$ and for various values of $s_{0}$. Therefore, in Fig. 5, we show the numerical evaluation of the asymptotic value $r_{\infty }$ as a function of the parameter $R_{0}$ as calculated by means of the SEIR and the NRDE model by taking $\tau_{I} = 1.0$ both for $s_{0} = 0.980$ and for $s_{0} = 0.999$. By these curves we notice that indeed the output for the SEIR and the NRDE model juxtapose, and for $R_{0} > 2$ all points tend to collapse into single spots. By these curves we notice that a rapid increase of $r_{\infty }$ occurs for $R_{0} > 1$, thus giving another way of recognizing the effects of an epidemic outbreak.

We now consider the duration $\tau_{D}$ of the disease. In order to make useful predictions, we introduce an empirical cutoff. In fact, in order to decide when the epidemic outbreak can be thought to be finished, we may establish that the quantity $i\left( \tau \right)$ lye below a small quantity $\varepsilon$, so that the duration $\tau_{D}$ is implicitly defined by the following condition:

17 $$ i\left( {\tau_{D} } \right) = \varepsilon . $$

**Fig. 6:**
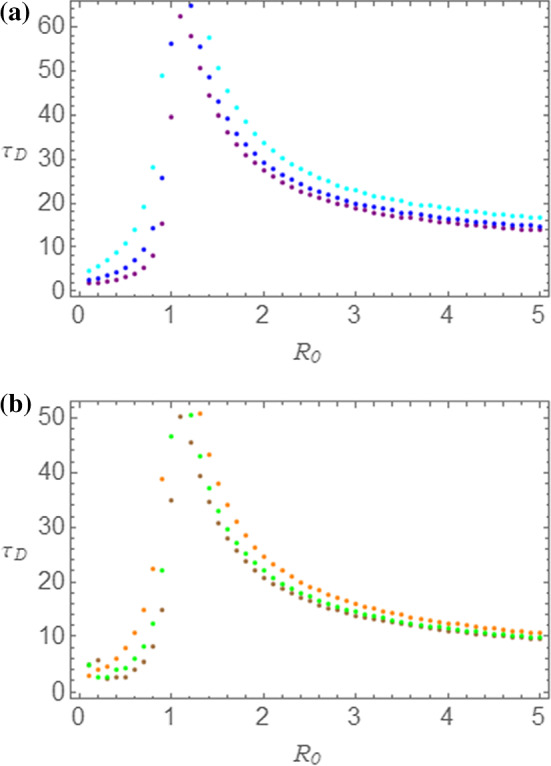
Duration $\tau_{D}$ versus $R_{0}$ of the disease obtained by means of the SEIR (**a**) and of the NRDE (**b**) models for $s_{0} = 0.995$ and $\tau_{I} = 1.00$. In panel (**a**) the correspondence between colors and $\varepsilon$ values is as follows: $\varepsilon = 0.0001$ (cyan); $\varepsilon = 0.0005$ (blue); $\varepsilon = 0.0010$ (purple). In (**b**) the correspondence between colors and $\varepsilon$ values is as follows: $\varepsilon = 0.0001$ (orange); $\varepsilon = 0.0005$ (green); $\varepsilon = 0.0010$ (brown)

We can numerically determine the duration of the epidemic regime as predicted by the SEIR and NRDE models as a function of the parameter $R_{0}$. Let us then set $m_{0} = 0.005$ ($s_{0} = 0.995$) and $\tau_{I} = 1.0$. Before making a comparison between the SEIR and the NRDE models, in Fig. 6a, b, we investigate the outcome of the numerical evaluation of the $\tau_{D}$ versus $R_{0}$ dependence for $s_{0} = 0.995$ and $\tau_{I} = 1.00$ separately for the two models. In Fig. 6a, predictions obtained by the SEIR model have been reported for the following cutoff values: $\varepsilon = 0.0001$ (cyan); $\varepsilon = 0.0005$ (blue); $\varepsilon = 0.0010$ (purple). In Fig. 6b, on the other hand, predictions obtained by the NRDE model have been reported for the following cutoff values: $\varepsilon = 0.0001$ (orange); $\varepsilon = 0.0005$ (green); $\varepsilon = 0.0010$ (brown). From these curves, we can once more observe the rather clear distinction between two regions, the first below and the second above $R_{0} = 1.0$. In the first region, we notice a sharp increase of the duration $\tau_{D}$ for increasing value of $R_{0}$ up to a rather high cusp value. In the second region, on the other hand, an asymptotic decrease of $\tau_{D}$ for increasing value of $R_{0}$ is present. As for the dependence on the cutoff value $\varepsilon$ of the plots in Fig. 6a, b, we notice that lower values of $\varepsilon$ imply slightly higher values of $\tau_{D}$, as it could be expected. It is important to stress that the cutoff value $\varepsilon$, comparable with $i_{0}$, is a measure of the socially acceptable number of infectious individuals in a given sanitary system. In this way, the parameter $\varepsilon$ can be considered as a tolerable endemic threshold for the illness.

**Fig. 7:**
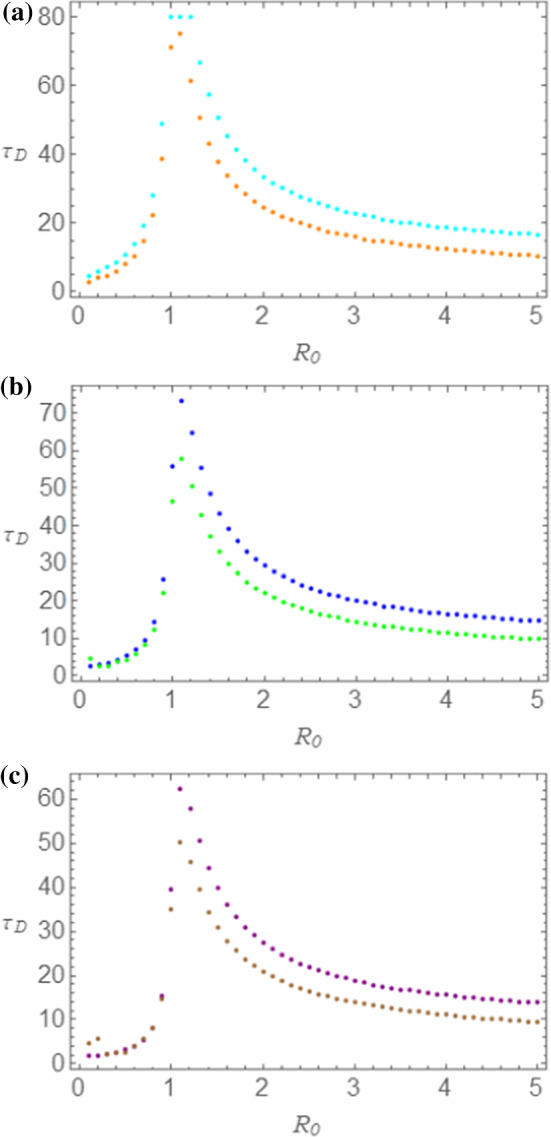
Comparison between the duration $\tau_{D}$ of the disease obtained by means of the SEIR and of the NRDE models for $s_{0} = 0.995$ and $\tau_{I} = 1.00$. In (**a**) $\varepsilon = 0.0001$; in (**b**) $\varepsilon = 0.0005$; in (**c**) $\varepsilon = 0.0010$. The choice of colors is the same as in Fig. 6a, b

In Fig. 7a–c we compare the behavior of $\tau_{D}$ versus $R_{0}$ predicted by the two models. The same choice of parameters and colors as in Fig. 6a, b has been made. In particular, in Fig. 7a–c, the $\tau_{D}$ versus $R_{0}$ plots are represented, in the order, for $\varepsilon = 0.0001$ (SEIR model in cyan; NRDE model in orange), for $\varepsilon = 0.0005$ (SEIR model in blue; NRDE model in green), for $\varepsilon = 0.0010$ (SEIR model in purple; NRDE model in brown). Coherently with what observed when we have considered the dynamics of the number of infectious individuals, the $\tau_{D}$ versus $R_{0}$ plots obtained by means of the NRDE model lye below those obtained by the SEIR model, meaning that the duration of the disease predicted by the NRDE model is lower than the corresponding quantity predicted by the SEIR model.

## Oscillations in the number of infectious individuals

In the present section, we give an account of the oscillating pattern, superimposed to the bell-shaped profile, in the initial part of the curve of infectious individuals in the NRDE model. We start by considering Eq. (15a) and set $s\left( \tau \right) = 1 - m\left( \tau \right) \approx 1$, so that:

18 $$ \frac{\text{d}}{{{\text{d}}\tau }}m\left( \tau \right) \approx R_{0} \left[ {m\left( {\tau - \tau_{I} } \right) - m\left( {\tau - \tau_{I} - 1} \right)} \right] $$

This assumption is justified by the fact that, being in general very low the value of $m_{0} = i_{0} = 1 - s_{0}$, the number of susceptible individuals can be initially taken almost equal to $N$, so that $s\left( \tau \right) \approx 1$, as set above. Let us now assume that the initial form of the function is given by an oscillating pattern superimposed to an exponential growth (we shall only take into account the epidemic regime), so that we set:

19 $$ m\left( \tau \right) \approx A e^{{\left( {\alpha + i\omega } \right)\tau }} + Be^{\beta \tau } . $$

where $A$, $\alpha$, $\omega$, $B$, $\beta$ are real constants. By substituting the above expression in Eq. (16), we have:

20 $$ A\left( {\alpha + i\omega } \right) + B\beta e^{\beta \tau } \approx R_{0} Ae^{{ - \left( {\alpha + i\omega } \right)\tau_{I} }} \left[ {1 - e^{{ - \left( {\alpha + i\omega } \right)}} } \right] + R_{0} Be^{\beta \tau } e^{{ - \beta \tau_{I} }} \left[ {1 - e^{ - \beta } } \right]. $$

By now equating the factors of the coefficients $A$ and $B$, we obtain the following two equations:

21a $$ \frac{{\left( {\alpha + i\omega } \right)}}{{R_{0} }}e^{{\left( {\alpha + i\omega } \right)\tau_{I} }} \approx 1 - e^{ - i\omega } ; $$

21b $$ \frac{\beta }{{R_{0} }}e^{{\beta \tau_{I} }} \approx 1 - e^{ - \beta } . $$

Therefore, if the above set of transcendental equations, for given values of $R_{0}$ and $\tau_{I}$, has one or more solutions for $\omega > 0$, the curves in the NRDE model will show initial oscillations. It is a rather formidable task to provide an analytic solution of the Eqs. (21a, b). However, we may attempt to solve these equations by considering $\alpha$ very small, the latter being an assumption consistent with numerical solutions. In this way, by setting $\alpha = 0$ in Eq. (21a), we have:

22 $$ \frac{i\omega }{{R_{0} }}e^{{i\omega \tau_{I} }} \approx 1 - e^{ - i\omega } . $$

By separating the real and imaginary parts in Eq. (22), we have:

23a $$ \sin \omega = \frac{\omega }{{R_{0} }}\cos \omega \tau_{I} . $$

23b $$ \cos \omega = 1 + \frac{\omega }{{R_{0} }}\sin \omega \tau_{I} . $$

By squaring both members in Eqs. (23a, b) and by summing homologous sides of these equations, we obtain:

24 $$ \frac{\omega }{{R_{0} }}\left( {\frac{\omega }{{R_{0} }} + 2\sin \omega \tau_{I} } \right) = 0. $$

It is here worth to mention that non-trivial solutions of Eq. (24) are possible if the condition $\tau_{I} \ne 0$ is respected. This observation implies the absence of oscillations in the NRD model as confirmed by numerical simulations (not reported). Discarding the trivial solution $\omega = 0$ in Eq. (24), we focus our attention on the nonzero solutions and write:

25 $$ \frac{{\sin \omega \tau_{I} }}{{\omega \tau_{I} }} = - \frac{1}{{2R_{0} \tau_{I} }} \equiv - \gamma , $$

where the constant $\gamma$ is implicitly defined in Eq. (25). The properties of the oscillating function $\frac{\sin x}{x}$ are well-known in optics [19]. In analyzing this function, we notice that solutions to Eqs. (25) can be found if the following condition is satisfied:

26 $$ \gamma < \gamma_{0} \approx 0.2172, $$

where $- \gamma_{0}$ is the absolute minimum of the function $\frac{\sin x}{x}$. By Eqs. (25) and (26), we obtain the following threshold condition on $\tau_{I}$

27 $$ \tau_{I} > \frac{1}{{2\gamma_{0} R_{0} }}. $$

**Fig. 8:**
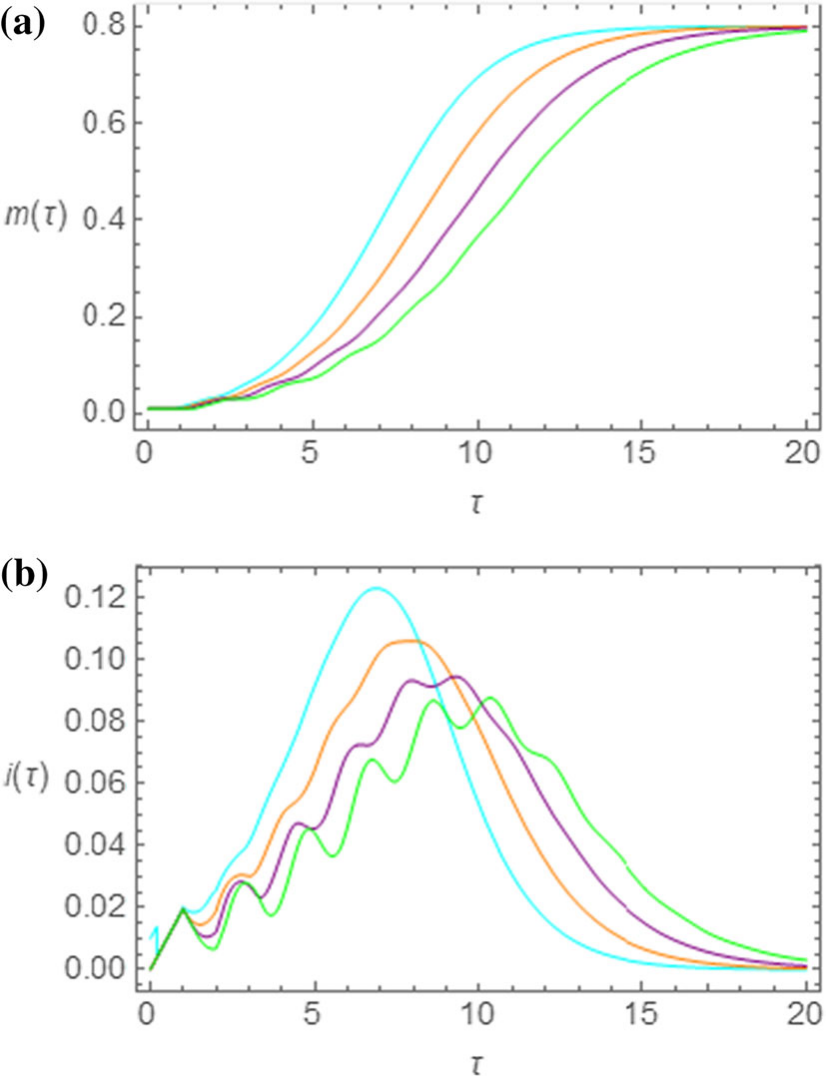
Time evolution of the functions $m\left( \tau \right)$ (**a**) and $i\left( \tau \right)$ (**b**), obtained by means of the NRDE model for $s_{0} = 0.99$ and $R_{0} = 2.00$. The incubation period has the following duration: $\tau_{I} = 0.80$ (cyan curve); $\tau_{I} = 1.00$ (orange curve); $\tau_{I} = 1.20$ (purple curve); $\tau_{I} = 1.40$ (green curve)

The above relation is an interesting result, by which we conclude that oscillatory phenomena in the time evolution of the function $i\left( \tau \right)$ are not present if $\tau_{I}$ is lower than the threshold value $\tau_{I}^{\left( c \right)} = \frac{1}{{2\gamma_{0} R_{0} }}$. Let us now take $R_{0} = 2.0$ and let us investigate the behavior of the functions $m\left( \tau \right)$ and $i\left( \tau \right)$ as $\tau_{I}$ goes through the critical value $\tau_{I}^{\left( c \right)}$ from below. We thus expect that the curves shown for $\tau_{I} < \tau_{I}^{\left( c \right)} \approx 1.151$ will not show marked oscillatory behavior, contrarily to those obtained for $\tau_{I} > \tau_{I}^{\left( c \right)}$. Therefore, in Fig. 8a, b we report the time dependence of the quantities $m\left( \tau \right)$ and $i\left( \tau \right)$, respectively, for $s_{0} = 0.99$ and $R_{0} = 2.0$. While the precursors of the oscillatory pattern are less evident in the cumulative function $m\left( \tau \right)$ for $\tau_{I} = 1.20, 1.40$, shown in Fig. 8a as purple and green curves, respectively, oscillations are rather pronounced in Fig. 8b, where the function $i\left( \tau \right)$ is shown for the same values of $\tau_{I}$ with the same color choice. On the other hand, the orange curves in Fig. 8a, b are only slightly undulated, since $\tau_{I} = 1.00 < \tau_{I}^{\left( c \right)}$. Finally, the cyan curves are almost free of oscillations, given that $\tau_{I} = 0.80$.

Having illustrated the properties of the solution of Eq. (21a) under the assumption of very small decay of the oscillatory pattern, we now proceed by solving Eq. (21b) numerically, by means of a rather elementary algorithm, which we shall here illustrate. We first solve Eq. (21b) for the parameter $R_{0}$ in terms of $\beta$ and of the parameter $\tau_{I}$. We then solve the same Eq. (21b) for the parameter $\tau_{I}$ in terms of $\beta$ and of the parameter $R_{0}$. In this way, we obtain the following two functions:

28a $$ R_{0} = \frac{\beta }{{1 - e^{ - \beta } }}e^{{\beta \tau_{I} }} ; $$

28b $$ \tau_{I} = \frac{1}{\beta }\ln \left( {\frac{{1 - e^{ - \beta } }}{\beta }R_{0} } \right). $$

**Fig. 9:**
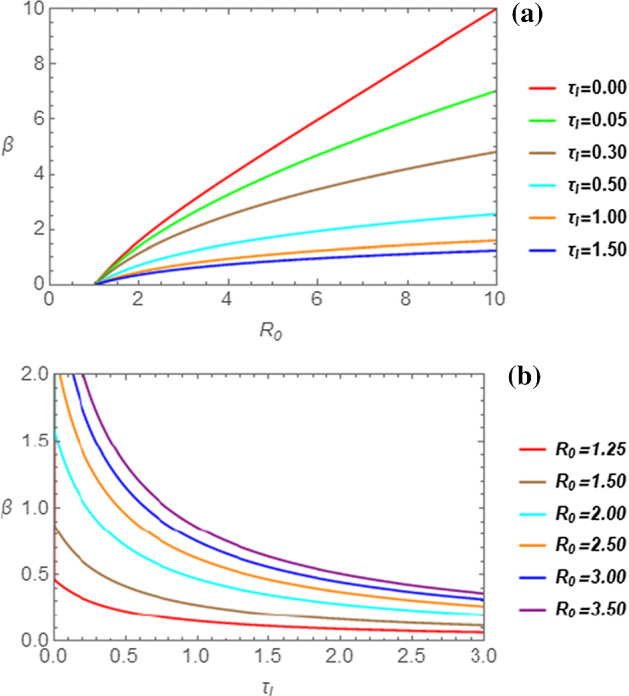
In (**a**) the parameter $\beta$ is shown as a function of $R_{0}$ for the following values of $\tau_{I}$ (top to bottom): 0.00, 0.05, 0.30, 0.50, 1.00, 1.50. In (**b**) the parameter $\beta$ is shown as a function of $\tau_{I}$ for the following values of $R_{0}$ (bottom to top): 1.25, 1.50, 2.00, 2.50, 3.00, 3.50

We restrict our analysis to cases with $\beta > 0$, so that, by Eq. (28a), we obtain $R_{0} > 1$, coherently with the hypothesis of epidemic regime. Therefore, we first find the $R_{0}$ versus $\beta$ dependence numerically by Eq. (28a), obtaining a finite set of values $\left( {\beta_{k} ,R_{0k} } \right)$. It is then only a matter of exchanging the position of $\beta_{k}$ and $R_{0k}$ in the table to get the inverse relation $\left( {R_{0k} ,\beta_{k} } \right)$. This dependence is shown in Fig. 9a for various value of $\tau_{I}$. All curves in Fig. 9a originate at $R_{0} = 1$, since we have chosen to only consider epidemic outbreaks. The parameter $\tau_{I}$ acts as a moderating factor in the dynamics of the disease at an initial stage of the epidemic outbreak. In fact, the values of $\beta$ tend to decrease as $\tau_{I}$ increases, since the curve in Fig. 9a tend to flatten for increasing values of $\tau_{I}$.

Following a similar reasoning, we obtain the curves shown in Fig. 9b. This time we need to consider Eq. (28b) and set $\tau_{I} > 0$, since the disease cannot appear before the contact between an infectious and a susceptible individual takes place. Therefore, in order to fulfil this requirement, by Eq. (28b) we set:

29 $$ \frac{\beta }{{1 - e^{ - \beta } }} < R_{0} . $$

Taking into account this constraint and applying the same procedure indicated in getting the $R_{0}$ versus $\beta$ plots, we obtain the curves shown in Fig. 9b. Naturally, we once again recover the fact that, at fixed values of $R_{0}$, the quantity $\tau_{I}$ acts as a moderating factor in the exponential growth of the cumulative function $m\left( \tau \right)$.

We may here mention that the observed oscillatory patterns in the curves in Fig. 8b does not arise from stochastic or seasonal effects, as in Ref. [4], but they are intrinsic self-sustained phenomena originated by the interplay between the two delay times present in the model. Moreover, the memory effects introduced in the present work are rather different from those considered in Ref. [4]. In fact, in the latter work memory effects originate from introducing the notion of fractional derivative into the dynamics of a SIR-type model. Therefore, while the memory effects in the NRDE model are determined by the illness characteristic times, i.e., the recovery time $t_{R}$ and the incubation time $t_{I}$, fractional derivative based models induce memory effects which are not directly related to these characteristic times. Despite these differences, it could be useful to introduce, in continuous retarded compartmental models, the seasonality properties of infectious diseases considered in Ref. [4].

## Conclusion

The NRD model [15], describing the time evolution of non-lethal infectious diseases in a fixed-size population of $N$ individuals, has been reconsidered by introducing a quiescent time $t_{I}$. In this way, an NRDE model is obtained from which the NRD model can be recovered by setting $t_{I}$ equal to zero. Therefore, the NRDE model can be considered as a non-trivial extension of the NRD model, in much the same way the SEIR model generalizes the SIR model.

We have found that the period of incubation of the illness provides new features in the dynamics of the infectious diseases with time delay. In fact, it is seen to affect the duration of the disease and the initial exponential growth of the cumulative function $m\left( \tau \right)$ during an epidemic outbreak.

When comparing the NRDE model to the SEIR model, analogies and differences between these two deterministic compartmental models are detected. The main analogy is the estimate of the asymptotic value $r_{\infty }$ of the curves describing the ratio $r\left( \tau \right)$. In fact, despite the different dynamic evolution of these curves, the quantity $r_{\infty }$ remains unaltered for epidemic outbreaks for all values of $\tau_{I}$. This feature is also shared by the SIR and the NRD models. As far as the dependence on $R_{0}$ of the duration $\tau_{D}$ of the disease is concerned, both the SEIR and the NRDE models predict similar patterns, even though the NRDE estimate of $\tau_{D}$ is slightly lower than the value predicted by the SEIR model. Among other analogies we may also mention the qualitative overall behavior of the curves describing the time evolution of the number of susceptible, infectious, and recovered individuals.

Interestingly, we have found that an oscillatory pattern superimposed to the bell-shaped profile of the $i\left( \tau \right)$ curves is a distinctive feature of the NRDE model. Self-sustained oscillations are an important dynamical fingerprint of the model, deserving further attention. Indeed, appearance of oscillations in epidemic records can be attributed to spurious effects or can be confused with the effects of lockdown measures, the latter playing the role of an external time-dependent driving for the dynamical system. Systems displaying self-sustained oscillations can evidence complicated response to a time-dependent driving. In fact, forcing terms can stabilize oscillating solutions also when they are not expected in static conditions. These arguments are to be taken into account in order to design optimal strategies to mitigate the effects of the epidemic evolution. The effectiveness of such strategies, which are studied by system control theory, is strongly dependent on the specific epidemic model considered. Deterministic compartmental models are the natural testbed to implement optimal strategies. In view of the dynamical richness of NRD and NRDE model, the interplay between time-dependent forcing terms and memory effects (originated by the characteristic delay times) could force to modify the optimality criteria of epidemic containment strategies, as done, for example, in Ref. [4]. In view of its relevance, the forced response of delay epidemic models needs to be investigated in future works.
